# Comprehensive Review of Pulmonary Hypertension and Treatment Options in the Paediatric Population

**DOI:** 10.7759/cureus.30622

**Published:** 2022-10-23

**Authors:** Pragyna D Parmita, Archana R Thool

**Affiliations:** 1 Medicine, Jawaharlal Nehru Medical College, Datta Meghe Institute of Medical Sciences, Wardha, IND; 2 Ophthalmology, Jawaharlal Nehru Medical College, Datta Meghe Institute of Medical Sciences, Wardha, IND

**Keywords:** congenital heart disease, thromboembolic disease, septostomy, endarterectomy, pulmonary hypertension

## Abstract

Pulmonary hypertension (PH) is a complex condition that can occur as a result of a wide range of disorders, including left heart disease, lung disease, and chronic pulmonary thromboembolism. Multiple improvements have been made in the diagnosis and treatment of pulmonary arterial hypertension (PAH) including a greater understanding of the involvement of extrapulmonary vascular organ systems, validated point of care, clinical assessment tools, and a focus on the initial exposure of numerous pharmacotherapeutics in the appropriate level of care. To achieve a minimal symptom burden, improve the patient's biochemical, hemodynamic, and functional profile, and reduce adverse impact, early diagnosis of PAH is a key objective today. The preferred method of management for thromboembolic PH, which is chronic, is pulmonary endarterectomy since the majority of affected patients are operable. The timing of pulmonary endarterectomy should never be delayed for medical reasons, and risk stratification can enable us to select patients who have a high chance of success. Patients who are not qualified for endarterectomy should be referred for drug trials. Even though there are more effective ways to guarantee a sufficient, long-lasting septostomy, atrial septostomy is promising but undervalued. The procedure's indications remain the same and need to be taken into account more frequently. Class III or IV patients who are not improving need to be consulted at a transplant centre as soon as possible as they may be candidates for potential recipients of bilateral sequential lung or heart-lung transplants, which is a significant choice for some people.

PH is rarely linked to other conditions like connective tissue or thromboembolic disease. It is either idiopathic or linked to congenital heart disease. Infants and children with PH are more frequently recognised in conjunction with a congenital diaphragmatic hernia and developmental lung diseases like bronchopulmonary dysplasia. Although the underlying disease has not yet been treated and advanced structural changes have not yet been reversed, the value of natural life and survival have suggestively increased. Children's haemodynamic and functional outcomes have improved as a result of endothelin receptor antagonists, prostacyclin analogues, and phosphodiesterase type 5 inhibitors, which are examples of targeted pulmonary vasodilator therapies. The health maintenance of paediatric PH is still difficult because treatment decisions are largely based on the findings of adult studies that have been supported by evidence and the clinical expertise of paediatric specialists.

## Introduction and background

Pulmonary hypertension (PH) is a constellation of diseases that shares signs and symptoms of dyspnoea, fatigue, chest pain, palpitations, and syncope. The interruption of blood flow to our heart and lungs caused by PH poses a risk. Pulmonary arteries narrow as a consequence of high blood pressure in these arteries. To deliver oxygen-depleted blood to our lungs, our hearts must work even harder [1]. The prevalence of pulmonary arterial hypertension (PAH) rises with age and is higher in women. Significant growth has emerged in the underlying causes, impacts, and treatments of PAH, a condition with many different aetiologies. Given their increased risk for speedy deterioration, people having PAH require constant observation and frequent evaluations. If these needs are met, prompt treatment intensification can be initiated to slow the development of the condition [2]. Numerous PH expert centres have started using telemedicine techniques to assess potentially complicated patients in certain circumstances. Finding the cause of PH in patients can be difficult, yet it is crucial because the available management options are influenced heavily by the aetiology of the disease. Telehealth played a significant but supporting role in ambulatory health care [3]. Right ventricular failure (RVF), the far more prevalent cause of death in PAH, is aided by obstructive, hyperproliferative, vascular lesions, vasoconstriction of pre-capillary arterioles, and venous obstruction [4].

Patients present with increased pulmonary vascular resistance (PVR), and right ventricular afterload, which leads to RVF [2]. Pulmonary vasodilators are the major PAH treatments presently offered. They minimize symptoms and drastically cut on hospital admissions, but they are expensive and ineffective. It is critical to obtain a diagnosis and begin treatment for PH as soon as possible because it can influence the entire body. The treatment regimen that the doctor recommends will depend on the root cause of PH. Untreated PH is potentially lethal irrespective of the cause [4,5]. Due to a decrease in peripheral vascular resistance, the pulmonary arterial pressure (PAP), which is equal to the systemic pressure in utero, decreases after birth and attains adult levels by two to three months of age. Adults, children, and term infants that have an elevated PAP and a pulmonary artery wedge pressure of less than 15 mmHg at sea level are referred to as having PAH. In addition to indexed pulmonary vascular resistance (PVRI), the criteria for infants and children also take into consideration specific classes of paediatric PH that cannot be recognized by mean pulmonary arterial pressure (mPAP) alone [5]. Despite having elevated mPAP, children with left-to-right shunts (aortopulmonary or intracardiac shunts) may not develop pulmonary hypertensive vascular disease (PHVD) in their early years. On the other hand, kids without a subpulmonary ventricle could still have PHVD even with an mPAP of less than 25 mmHg. Complex underlying aetiologies and frequent comorbidities, such as prematurity, neonatal lung diseases (bronchopulmonary dysplasia (BPD)/chronic lung disease (CLD), lung hypoplasia), chromosomal anomalies, and polymalformation syndrome, are typical management challenges for paediatric PH. Paediatric PAH is linked to stunted growth, especially in younger kids (zero to five years old) and those with PAH and congenital heart disease (CHD). Such failure to thrive is linked to a higher risk of death [5,6].

## Review

Methodology

A systemic search on electronic databases (PubMed, Web of Science, and Google Scholar) was initiated. Articles on the comprehensive review of pulmonary hypertension and treatment options in the paediatric population were explored using the following keywords: pulmonary hypertension, thromboembolic disease, dyspnoea, and right heart catheterization. Around 100 articles were found relevant to the studies and screened for inclusion and exclusion criteria. Original articles and review articles published in the English language only were included in the study. Finally, around 39 references were imported to Zotero (Roy Rosenzweig Center for History and New Media, Fairfax, Virginia) and cited in this review article.

Aetiopathogenesis

**Figure 1:**
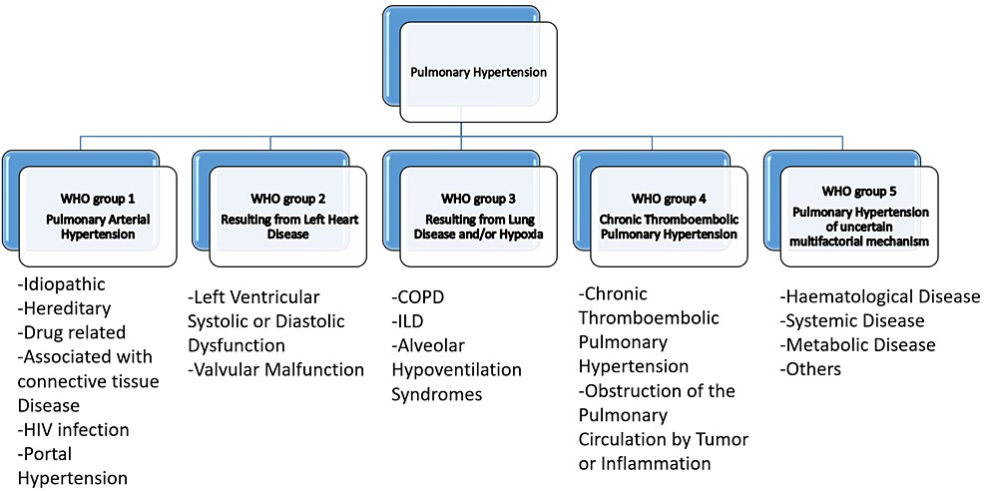
Comprehensive clinical classification of pulmonary hypertension according to the World Health Organization functional class Adapted from [7]. WHO: World Health Organization; HIV: human immunodeficiency virus; COPD: chronic obstructive pulmonary disease; ILD: interstitial lung disease.

PAH may be idiopathic or secondary to a number of conditions, but regardless of the underlying aetiology, patients display similar pathological changes, which include increased pulmonary arteriole contractility, endothelial dysfunction, remodelling and proliferation of both endothelial and smooth muscle cells, and in situ thrombi [5]. Pathologies including diffuse alveolar epithelium loss, capillary damage/haemorrhage, hyaline membrane formation, alveolar septal fibrous proliferation, and pulmonary consolidation are believed to be the most common in the lung [6]. A resting mPAP of +/> 20 mmHg is the only haemodynamic indicator of pulmonary hypertension, which is not a diagnosis in and of itself. Figure 1 represents the comprehensive clinical classification of pulmonary hypertension, according to the World Health Organization functional class.

PAH can advance in both men and women, which results in right heart failure and mortality due to increased pulmonary vascular resistance [8]. Progressive pulmonary vascular defects are due to the disruption of three key signalling pathways: nitric oxide (NO), prostacyclin (PGI2) and thromboxane A2 (TXA2), and endothelin-1 (ET-1) [9]. In general, cyclooxygenase-2 dysregulation, reduced PGI2 production, and NO synthase (eNOS) dysfunction all contribute to PAH, which also ultimately results in concurrent vasoconstrictive and mitogenic effects from an upregulated ET-1 signalling system [10]. The enzyme eNOS, which generates nitric oxide in endothelial cells, catalyses the conversion of L-arginine to L-citrulline in the presence of oxygen, nicotinamide adenine dinucleotide phosphate (NADPH), and other cofactors. It also converts guanosine triphosphate (GTP) to cyclic guanosine monophosphate (cGMP). Reduced nitric oxide bioavailability in PAH results in vasoconstriction, an increase in smooth muscle cell proliferation, inflammation, and thrombosis, as well as decreased nitric oxide bioavailability [11]. Arachidonic acid is converted into prostacyclins in endothelial cells by the enzymes cyclooxygenase and prostacyclin synthase. Adenylate cyclase is activated when PGI2 binds to I-prostanoid (IP) receptors in smooth muscle cells. Adenosine triphosphate (ATP) is transformed by this enzyme into cyclic adenosine monophosphate (cAMP), which leads to smooth muscle relaxation and subsequent vasodilation. Prostacyclin has anti-inflammatory and antithrombotic properties. It also reduces smooth muscle proliferation and platelet aggregation. A peptide known as ET-1 functions as a strong vasoconstrictor. Endothelin-converting enzymes synthesize ET-1 on the membranes of endothelial cells from the precursor peptide big-endothelin-1. ETA and ETB, two G-protein coupled receptors, are activated by ET-1. When ETA is activated, it promotes vasoconstriction, hypertrophy, proliferation, cell migration, and fibrosis. ETA is found on vascular smooth muscle cells. Both the surface of endothelial cells and vascular smooth muscle contain ETB. When ETB is activated, smooth muscle experiences vasoconstriction, while endothelial surfaces experience vasodilation and anti-proliferation when nitric oxide and prostacyclin production are activated [12]. Elevated pulmonary vascular resistance, which raises right atrial wall stress in sufferers with pulmonary hypertension, can cause right atrial dilatation, right atrial dysfunction, and right atrial fibrillation. The right ventricle can adjust by thickening its walls and becoming more contractile as the pressure load slowly rises. Thus, while preserving the right ventricle output, the right ventricle starts pumping from low pressure to high pressure. However, the right ventricle cannot continue to remodel; therefore, if the high pulmonary vascular resistance continues, the right ventricle will dilate to keep the stroke volume constant [13].

Clinical presentation

Angina, exertional dyspnoea, exhaustion, feebleness, presyncope, and syncope are all typical signs of pulmonary hypertension [14]. With progressive right ventricular failure, fluid administration needs close monitoring as it may cause abdominal distention and pedal oedema due to progressive right ventricular failure. Hepatomegaly, ascites, peripheral oedema, a tricuspid regurgitant murmur, an augmented second heart sound, a third heart sound from the right ventricle, raised jugular venous pressure having an uncharacteristic waveform, low-volume arterial pulses, and left parasternal lift or retraction are all possible physical findings [14]. Because the clinical supervision of pulmonary arterial hypertension in children is frequently non-specific, diagnosing the condition may be challenging. Infants with pulmonary hypertension frequently experience tachypnea, irritability, and failure to thrive due to low cardiac output, though the elderly may experience workout intolerance and rarely adult-like chest pain. Dyspnoea with strain is the most common presenting indication [15].

Diagnosis and investigations

Electrocardiography

The methodical and thorough diagnostic methodology is essential to arrive at a precise analysis and treatment strategy because the aetiology of pulmonary hypertension is extremely diverse. Furthermore, idiopathic pulmonary arterial hypertension (IPAH) can only be diagnosed "per exclusion", that is, by ruling out all other known causes of pulmonary hypertension [16]. ECG and echocardiography are used to screen patients for pulmonary hypertension. A chest X-ray and/or chest computed tomography (CT) must be reflected if these findings point to PH/PHVD. Cardiac catheterization may be delayed and pharmacotherapy, including intravenous prostanoids, should be started immediately if PH/PHVD is unembellished and the patient is critical with heart failure and/or pulmonary vascular crisis [17].

The incidence of one or more tenacious perfusion defects on ventilation-perfusion skimming is the primary diagnostic indicator. Haemodynamic measurements aid diagnosis and perioperative danger assessment. Right heart catheterization is the gold standard for the diagnosis of pulmonary hypertension [18]. No diagnosis is excluded by a normal ECG of pulmonary hypertension, but it may provide supporting evidence of the condition. In contrast to mild pulmonary hypertension, severe pulmonary hypertension is more likely to have an abnormal ECG, including P pulmonale, right axis deviation, right ventricular hypertrophy, right ventricular strain, right bundle branch block, and QTc prolongation, which are examples of ECG abnormalities. Right ventricular strain is more sensitive than right ventricular hypertrophy, which has insufficient sensitivity (55%) and specificity (70%) to be used as a screening tool [19]. ECG has been regarded as a trustworthy indicator of PH. Certain ECG characteristics worsen the prognosis of patients with PAH [20]. Advanced disease may result in supraventricular arrhythmias, particularly atrial flutter and atrial fibrillation, with a cumulative incidence of 25% of patients after five years [21].

Chest Radiograph

Chest radiographs are unusual in 90% of IPAH patients during the analysis period [22]. The central pulmonary arterial dilatation seen in people with pulmonary hypertension shows disparity with the "pruning" of the peripheral blood vessels. In more severe cases, the right atrium and right ventricle might enlarge. By displaying signs of lung disease, a differential analysis of pulmonary hypertension may be aided by a chest radiograph. Chest radiography helps in differentiating between arterial and venous pulmonary hypertension by respectively determining increased and decreased artery-to-vein ratios [23].

Pulmonary Function Tests and Arterial Blood Gases

The impact of essential airway or parenchymal lung ailment is determined by pulmonary function assessments and arterial blood gas measurements. Rendering to the sternness of the ailment, people with pulmonary arterial hypertension typically have a slight to reasonable decrease in lung volume. High-resolution CT could be used to assess the sternness of interstitial lung sickness and emphysema. Emphysema and pulmonary fibrosis could end up causing spirometry to seem so pseudonormal; however, the diffusing capacity of the lungs for carbon monoxide is nearly always reduced, highlighting the importance of construing pulmonary function and lung imaging. In PAH, nocturnal hypoxaemia and central sleep apnoeas are indeed very common. Despite the possibility of normal diffusion capacity in pulmonary hypertension, maximum patients have reduced lung carbon monoxide diffusion capacity (DLCO). An unfavourable outcome is linked to an abnormally low DLCO, defined as 45% of predicted [24,25]. Scleroderma-associated PAH and parenchymal lung disease are two possible ways for the differential diagnosis of a low DLCO in PAH. Peripheral airway obstruction is unusual whereas airflow obstruction is frequent. Alveolar hyperventilation induces arterial carbon dioxide pressure to decrease, whereas the arterial oxygen pressure remains the same or is marginally lower than standard while at rest. Even before hypoventilation or obstructive sleep apnoea syndrome is presumed, an abrupt oximetry check or polysomnography should be accomplished. Severe exertional hypoxemia or a marked decrease in DLCO (60% of predicted) may be indicators of pulmonary venous-occlusive sickness or pulmonary capillary haemangiomatosis [26].

Echocardiography

Echocardiography is performed every time pulmonary hypertension is suspected. It can also be implemented to deduce the judgement of pulmonary hypertension in patients whose numerous echocardiographic findings are consistent. Echocardiography by itself is insufficient to support a treatment choice when treating pulmonary hypertension as a standalone condition; cardiac catheterization is necessary instead. Documents provided and/or endorsed by the European Association of Cardiovascular Imaging comprise stringent guidance describing the echocardiographic assessment of the right heart [27].

Cardiopulmonary Exercise Testing and Cardiac Magnetic Resonance Imaging

Cardiopulmonary exercise testing can be done non-invasively or in conjunction with haemodynamic testing for diagnostic purposes. Retrograde flow significantly reduced pulmonary arterial distensibility and late gadolinium enhancement seems to be strongly prognostic of the occurrence of pulmonary hypertension in patients with suspected pulmonary hypertension; nevertheless, no single cardiac magnetic resonance measurement can control pulmonary hypertension. If echocardiography is unclear in pulmonary hypertension patients, cardiac magnetic resonance may be beneficial in suspected congenital heart defect cases. In clinical situations such as suspected chronic embolism in young patients and pregnant patients, or when iodine-based contrast media injection is contraindicated, contrast-enhanced and unenhanced magnetic resonance angiography have a potential for the study of the pulmonary vasculature in patients with suspected chronic thromboembolic pulmonary hypertension [28]. In patients with pulmonary hypertension, a cardiopulmonary exercise test (CPET) measures the grade of comparative hypoperfusion of the lung and the systemic movement during implementation [29]. Cardiac magnetic resonance is a suggested non-invasive imaging technique for managing pulmonary hypertension. Although no single cardiac magnetic resonance measurement can completely rule out pulmonary hypertension in patients with alleged pulmonary hypertension, the occurrence of late gadolinium improvement, decreased pulmonary arterial distensibility, and reversing flow have great prognostic values for their documentation [30,31].

Abdominal Ultrasound Scan

Abdominal ultrasound helps identify some of the medical entities linked to pulmonary hypertension. Although it cannot formally exclude portal hypertension, an abdominal ultrasound may confirm it. Portopulmonary hypertension (POPH) is a form of PAH associated with portal hypertension with or without underlying chronic liver disease. Measurement of the gradient between free and occluded (wedge) hepatic vein pressure at the time of right heart catheterization can reliably confirm or rule out portal hypertension. The count of a colour Doppler exam and contrast agents may increase diagnostic precision [32].

Biomarkers in Pulmonary Hypertension

Although numerous biomarkers are being studied in pulmonary hypertension, lone brain natriuretic peptide (BNP) and its N-terminal prohormone are frequently assessed for medical judgements. Markers could be classified into markers of vascular dysfunction, markers of inflammation, markers of myocardial stress, and markers of low carbon monoxide [33]. N-terminal prohormone and BNP levels are prognostic indicators at the time of diagnosis and during subsequent evaluations because they are correlated with myocardial dysfunction [34].

Treatment

**Figure 2:**
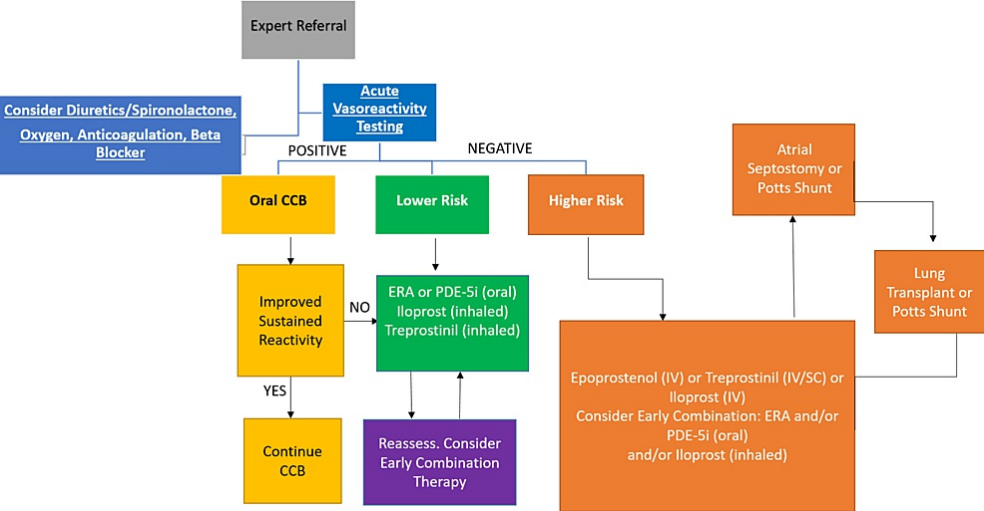
Evidence-based treatment modalities in pulmonary hypertension Adapted from [35]. CCB: calcium channel blockers; ERA: endothelin receptor antagonist; PDE: phosphodiesterase; IV: intravenous; SC: subcutaneous.

Patients in any group may require supportive therapies like diuretics, oxygen, and management of heart failure, including treatment of aggravating factors, optimization of fluid status, reduction of right ventricular afterload, and cardiac inotropes, if necessary, also in comorbidities such as sleep apnoea and chronic obstructive pulmonary disease. Figure 2 represents evidence-based treatment modalities in pulmonary hypertension.

In the above figure, the high-risk group refers to patients who present with syncope, show progression of symptoms, have significantly elevated levels of serum BNP, show severe right ventricle enlargement on echocardiography, and present with clinical evidence of right ventricle failure. Whereas low-risk group patients present with no syncope, do not show progression in their symptoms, and have minimally elevated serum BNP levels. Oral anticoagulation is recommended for patients suffering from pulmonary hypertension though patients do not have any past account of pulmonary embolism [35]. Due to the prevalence of iron deficiency in pulmonary hypertension, it is recommended to monitor iron levels and, if necessary, substitute iron (Figure 2) [35]. Oral beraprost, intravenous epoprostenol, intravenous and subcutaneous treprostinil, and iloprost, which comes in intravenous, oral, and nasal aerosol preparations, are all readily available prostacyclin analogues. An accurate oral non-prostanoid prostacyclin receptor agonist is selexipag. Endothelin's vasoconstrictive and mitogenic effects are prevented by endothelin receptor antagonists like bosentan, macitentan, and ambrisentan [36].

Surgical Therapy

Optical coherence tomography or intravascular ultrasound may be used in addition to each other, depending on local customs and practice. The last alternative in the investigative process is selective pulmonary angiography. It shows ring-like stenosis, nets (or "slits"), pouches, abnormalities in the wall, comprehensive vascular barriers, and bronchial collaterals. This image analysis helps with the technical operability evaluation. Several echocardiographic alternatives have been suggested as predictors in PAH but only the tricuspid annular plane systolic excursion (TAPSE) has been demonstrated to be strongly linked with improved existence throughout treatment, suggesting its latent usefulness as a management goal [37]. For most patients, symptoms are significantly relieved, and haemodynamics are almost back to normal. Treatment for chronic thromboembolic pulmonary hypertension requires a true bilateral endarterectomy through the medial layer of the pulmonary arteries, which might lead to circulatory arrest, lacking the necessity for cerebral perfusion, in disparity to surgical embolectomy for severe pulmonary embolism (PE) [38].

Medical Therapy

Anticoagulants, diuretics, and oxygen (O2) in the event of hypoxemia or heart failure constitute the best medical care. It is advised to take anticoagulants for the rest of one's life even though there are certainly no statistics on their effectiveness and care for novel oral anticoagulants. Pneumonia microvascular disease justifies the usage of medications permitted for pulmonary hypertension off-label [39]. Table 1 shows recommendations for the efficacy of drug monotherapy for pulmonary hypertension.

**Table 1:** Recommendations for efficacy of drug monotherapy for pulmonary hypertension Adapted from [39].

Agent	Dosage	Mechanism of action	Side effects	Cautions
Calcium channel blockers				
Nifedipine, amlodipine, diltiazem	Initial dosage: 0.6-0.9 mg/kg/day in 3 divided doses; maintenance dosage: 2-5 mg/kg/day (for nifedipine). Initial dosage: 1.5-2 mg/kg/day in 3 divided amounts; maintenance dosage: 3-5 mg/kg/day in 3 divided doses (for amlodipine). Initial dosage: 2.5-5 mg/day; maintenance dosage: 2.5-5 mg/kg/day twice daily (for diltiazem)	Pulmonary/systemic vasodilation	Headache, stiffness, dizziness, feeling tired, vomiting, oedema, itching, gum hyperplasia, bradycardia, and systemic hypotension	Raise the dose after a lower one. Use extended-release preparations if at all possible. Hypotension risk potential
Prostacyclin				
Epoprostenol, iloprost, treprostinil	Initial dosage: 1-3 ng/kg/min; maintenance dosage: 50-80 ng/kg/min (for epoprostenol). Initial dosage: 2.5 micro g per inhalation; maintenance dosage: 5 micro g intravenous (for iloprost). Initial dosage: 1.25 to 2 ng/kg/min; maintenance dosage: 50-80 ng/kg/min (for treprostinil)	Pulmonary/systemic vasodilation antiplatelet aggregation	Reddening, headache, vomiting, diarrhoea, pain in the foot, rash, hypotension, cough, pain in the head, flushing pain in the jaw, intravenous infusion. Pain at the infusion site comparable to epoprostenol might need an advanced dosage	Possible danger of hypotension and bleeding due to the drugs. Symptoms of an airway reaction, severe hypotension, and intravenous reactive airway symptoms
Phosphodiesterase type 5 inhibitor				
Sildenafil and tadalafil	Initial dosage: 0.5 mg/kg/dose; maintenance dosage: 1 mg/kg/dose, 1 mg/kg/day according to studies	Pulmonary vasodilation, inhibition of the vascular remodelling	Pain in the head, dizziness, peripheral swelling, dyspepsia, diarrhoea, back pain, disturbances in vision, analogous to sildenafil, no substantial effect on sight	Cautious chronic use in children below 17 years of age. Clinically non-significant modifications in co-managed bosentan or ambrisentan

## Conclusions

High levels of pulmonary arterial pressure and pulmonary vascular resistance cause pulmonary hypertension, which also manifests as advanced loss and hindrance of the pulmonary vascular bed, causing dysfunctioning of the right ventricle and eventually leading to right ventricular fibrillation. Through the advent of embattled pulmonary vasodilator medication rehabilitation over the past three eras, the extended tenure of survival of kids suffering from pulmonary hypertension has enhanced. Causes of pulmonary hypertension seen in children are cardiomyopathy, or a weak heart muscle, chronic blood clots, congenital diaphragmatic hernia, or a hole in the diaphragm, with resultant lung hypoplasia (abnormally small lungs), cystic fibrosis, and Down syndrome. Even though modern therapeutic approaches have significantly improved prognoses, pulmonary hypertension management is still difficult in the modern era. Meanwhile, adult remedies are evidence-constructed after randomized trials, and vasodilator therapies for youngsters are principally created based on an understanding or expert view. Growing collaboration has led to an increase in paediatric pulmonary hypertension multicentre trials, which could ultimately lead to better clinical endpoints and the development of targeted treatment approaches. Effective diagnosis and characterization of the numerous procedures of pulmonary hypertension require careful evaluation of the medical past, physical state, echocardiograms, and haemodynamic limitations. Victims with pulmonary hypertension or chronic thromboembolic pulmonary hypertension should be referred as soon as possible to a specialized pulmonary hypertension hub and have access to targeted pharmaceutical and surgical treatments. There is no clear process for obtaining a drug or biomarker, and the conversion of these lessons into clinically useful biomarkers and new therapeutic agents has been slow, regardless of the fact that basic science studies have clarified a number of significant molecular pathways that underlie PAH. Additionally, preclinical research is siloed, and researchers, which include own selves, regularly disregard or fail to take cognizance able to compete with or supplement disease theories. As a result, PAH is varyingly viewed as a genetic disorder, an epigenetic syndrome, an autoimmune inflammatory disorder, or a disease of disturbed mitochondrial dynamics and metabolism. Even though no single cardiac magnetic resonance measurement can rule out pulmonary hypertension, retrograde flow, reduced pulmonary arterial distensibility, and late gadolinium enhancement seem to be strongly predictive of pulmonary hypertension in patients with suspected pulmonary hypertension.
